# The bare necessities of plant K^+^ channel regulation

**DOI:** 10.1093/plphys/kiab266

**Published:** 2021-06-11

**Authors:** Cécile Lefoulon

**Affiliations:** Laboratory of Plant Physiology and Biophysics, Bower Building, University of Glasgow, Glasgow G12 8QQ, Scotland

## Abstract

Potassium (K^+^) channels serve a wide range of functions in plants from mineral nutrition and osmotic balance to turgor generation for cell expansion and guard cell aperture control. Plant K^+^ channels are members of the superfamily of voltage-dependent K^+^ channels, or Kv channels, that include the Shaker channels first identified in fruit flies (*Drosophila melanogaster*). Kv channels have been studied in depth over the past half century and are the best-known of the voltage-dependent channels in plants. Like the Kv channels of animals, the plant Kv channels are regulated over timescales of milliseconds by conformational mechanisms that are commonly referred to as gating. Many aspects of gating are now well established, but these channels still hold some secrets, especially when it comes to the control of gating. How this control is achieved is especially important, as it holds substantial prospects for solutions to plant breeding with improved growth and water use efficiencies. Resolution of the structure for the KAT1 K^+^ channel, the first channel from plants to be crystallized, shows that many previous assumptions about how the channels function need now to be revisited. Here, I strip the plant Kv channels bare to understand how they work, how they are gated by voltage and, in some cases, by K^+^ itself, and how the gating of these channels can be regulated by the binding with other protein partners. Each of these features of plant Kv channels has important implications for plant physiology.

## Teaser

While plant voltage-dependent K^+^ channels embody many of the characteristics common among animal and bacterial channels, their gating continues to challenge mechanistic understanding.

## Introduction

Potassium (K^+^) is the most abundant inorganic macroelement maintaining turgidity of the plant cell. As a positively charged species, it balances the negatively charged nucleic acids and proteins, and it serves as a cofactor for some enzymatic activities. The transport of K^+^ is also a significant factor contributing to charge balance in the transport of other solutes across the cell membrane. Consequently, the transport of K^+^ regulates cellular hydration and is a critical factor in cell expansion (Very and Sentenac, 2003; Wang and Wu, 2013).

Potassium transport through plasma membrane occurs through different types of transport systems, notably channels that can be highly selective for K^+^, like the voltage-gated (Kv) channels that we will describe in this review, or less specific for cations like cyclic nucleotide-gated channel (CNGC), and transporters that can operate as uniporters or symporters with the proton as a driver ion, like high-affinity K^+^ transporter (HKT) or high-affinity K^+^/K^+^ UPtake/K^+^ transporter (HAK/KUP/KT; Gambale and Uozumi, 2006; Waters et al., 2013; Santa-María et al., 2018; Dietrich et al., 2020). Channels are described as passive because they facilitate K^+^ flux down the electrochemical gradient (Kochian et al., 1985) and generally operate with low transport affinity (Epstein et al., 1963) even if the Kv channel AKT1 can take up K^+^ in the range between 10 and 100 µM (Spalding et al., 1999). The most prominent feature of Kv channels, however, is that their opening and closing are controlled by voltage.

Kv channels are not the only voltage-dependent channels in plants. CNGC channels and the vacuole TPC1 channel also show voltage sensitivity. Unlike plant Kv channels, however, they are not specific for K^+^ and may carry also Ca^2+^ and Na^+^ (Guo et al., 2016; Li et al., 2017).

In this review, I focus on plant Kv channels, their structures, and their functions. I begin with an explanation of how K^+^ is conducted through the channel pore and how the channels achieve a high degree of selectivity. Thereafter, I introduce the structural features of plant Kv channels, compare these among the superfamily of Kv channels, and describe the different types of plant Kv channels. A previous review (Jegla et al., 2018) addressed the evolution of Kv channels. Here I will focus on how Kv channels are regulated by voltage and our present understanding of how the gating by voltage is controlled. Finally, in closing, the review will address other proteins that regulate or affect gating.

## Plant Kv channels are highly selective for K^+^

Hille, in his 1978 review, noted that channels are enzymes and their substrates are ions. Indeed, from a thermodynamic standpoint, all transporters are enzymes that facilitate the conversion of solutes from one energetic state to another across a membrane. Binding affinity and ion specificity for a channel define their transport properties. Long before the crystal structure of any ion channel was available, electrophysiological and radiotracer flux studies provided key insights into how channels work. Beyond the concepts of their regulation, which I address in the subsequent sections, our understanding of ion permeation was well established early on. Hodgkin and Keynes (1955) showed that ions in a channel must penetrate in single file through a water-filled pore, and work in the early 1970s led researchers to understand that the ions must move through a series of energetic barriers and binding sites that provided selectivity while still enabling ions to cross the pore at rates very close to their free diffusion in solution (Bezanilla and Armstrong, 1972; Hille, 1973).

The presence of this selectivity filter in the K^+^ channel pore was confirmed by mutating crucial residues, leading to the recognition of a highly conserved motif for K^+^ selectivity (Heginbotham et al., 1992, 1994). Among others, these studies showed that swapping the selectivity motif of a highly selective K^+^ channel with the motif from a poorly selective K^+^ channel that is permeable to Na^+^ and K^+^ also swapped permeability. Furthermore, single mutations in the pore motif created channels that transport a range of different alkali ions with little or no selectivity for K^+^.

**Figure 1:**
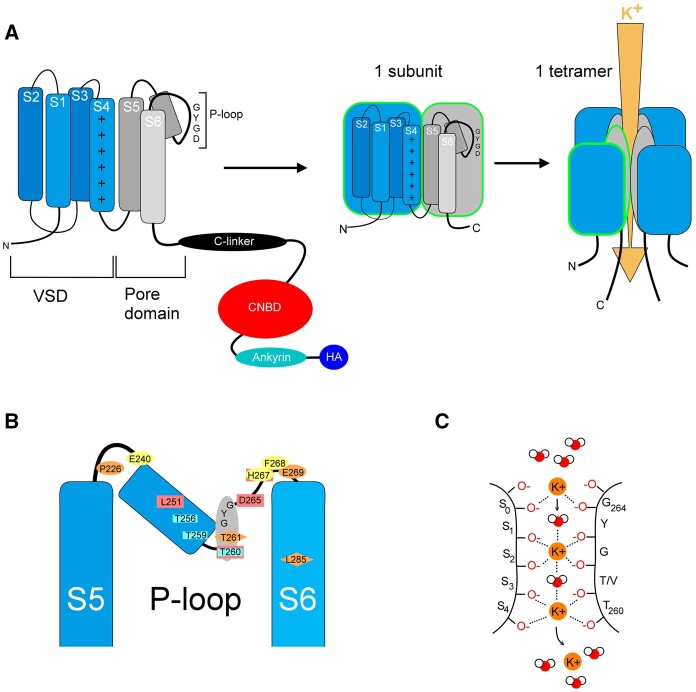
Potassium conduction through plant Kv channel. A, Plant Kv channel α-subunit domains (*left*) comprise the domains of the Voltage Sensing Domain (VSD, *blue*), pore bearing the selective TT/VGYG filter (*grey*), C-linker (*black*), CNBD (*red*), ankyrin domains (*turquoise*), and hydrophobic and acidic residues (*dark blue*). Four α-subunits assemble to form a functional K^+^ channel with the pore at the center (*right*). B, Schematic of the KAT1 pore domain and key amino acids, notably the TxGYG K^+^ permeability motif (*grey*), residues contributing to selectivity (*cyan*), and to Cs^+^ blockage (*red*). Sensitivity to K^+^ in gating (*orange*) is also associated with H^+^ sensitivity in the AKT2 channel (Geiger et al., 2002), whereas H^+^ sensitivity of KAT1 is separate (*yellow ovals*). Amino acids are found in AKT2 (*orange ovals*) and in SKOR and GORK (*orange diamonds*). C, Details of the K^+^ filter. The soft knock-on model is represented here. Motifs from two different subunits are shown, each bearing the TT(/V)GYG sequence. Two additional motifs (not shown) are positioned in front and behind the plane of the image, and contribute to the binding sites labeled here as S0 to S4. To permeate the selectivity filter, a K^+^ ion must be stripped of its aqueous shell, which is replaced by coordination with oxygen atoms of the backbone carbonyls groups of the TTGYG residues. In this model, each K^+^ ion is separated by a water molecule. The weak interactions between the oxygen atoms and K^+^ ions are indicated by the dotted lines.

We now know that Kv channels are formed by the assembly of four subunits, often referred to as α-subunits. In general, voltage-gated ion channels are comprised of four homologous domains although, unlike Kv channels, these domains are contained within a single polypeptide in mammalian Na^+^ and Ca^2+^ channels. At the center, the tetramer assembly forms a hydrophilic pore (Figure 1A) with each subunit contributing a domain that lines the pore. This pore domain is determined by two transmembrane α-helices, also referred to as Segments S5 and S6, linked together by a helical segment, the P-loop, that dips back toward the membrane (Figure 1). The P-loop contains the highly conserved TxGYG motif (TVGYG in *Arabidopsis thaliana* Kv channels but TTGYG in KAT1 and KAT2). This motif forms the K^+^ selectivity filter that is found in all Kv K^+^ channels, whether from plants, animals, or bacteria.

On entering the pore, the K^+^ ion engages with the pore and is stripped of its hydration shell to become coordinated by the carbonyl oxygen atoms of the channel TxGYG motif (Figure 1, B and C). The model proposed by Hodgkin and Keynes (1955) suggests that the K^+^ ions line up in a row and repulse one another so that each ion entering at one end of the pore “knocks” another ion out of the pore at the other end. Like newton balls, this "hard knock-on" model implies that the pore must be fully occupied by 4 K^+^ atoms before a K^+^ ion passes out of the pore at the far end. The theory is supported by molecular dynamic simulations (Köpfer et al., 2014), but crystal structures and more recent simulations suggest that one out of every two occupancy sites on the motif is filled by water and only two K^+^ ions need to occupy the pore at a time before an ion passes out at the far end (Berneche and Roux, 2001; Morais-Cabral et al., 2001; Kratochvil et al., 2016). This model is often cited as the “soft knock-on” process (represented in Figure 1C). Potassium ions have to line up to enter the pore as it is tight enough to let only one K^+^ atom at the time. Because the pore size closely matches the hydration space for a K^+^ ion, larger ions like Ca^2+^ cannot permeate the pore, and smaller ions like Na^+^ cannot enter because carbonyl oxygens of the filter are too far from each other to compensate for the dehydration of a Na^+^ ion. Consequently, Kv channels are around 20–50 times less permeant to Na^+^ than K^+^ (Thiel and Blatt, 1991; Schachtman et al., 1991; Müller‐Röber et al., 1995; Amtmann et al., 1997). Studies of the bacterial KcsA Kv channel show that the pore size and structure are maintained and strengthened by the interaction between amino acids located on the TxGYG motif and on the tilted pore helix on the P-loop (Doyle et al., 1998). This conjunction between the pore loop and the adjacent helices most likely explains why mutations in plant Kv channel TxGYG motif and in the pore helix will affect the ion selectivity of a channel. Mutated channels in these regions can become permeable to Rb^+^, Na^+^, and ${\text{NH}}_{4}^{+}$ (Figure 1B; Uozumi et al., 1995, Lacombe and Thibaud, 1998), or inhibition by Cs^+^ can be affected (Becker et al., 1996).

## Plant Kv channel diversity and structure

### Plant Kv channels are not Shaker but members of the CNBD channel subfamily

Although historically identified as Shaker channels, because Kv channels were first cloned from the *Shaker* mutant of fruit flies, plant Kv channels show the greatest similarity to the Kv subfamily of cyclic nucleotide-binding domain (CNBD) channels, and more specifically the KCNH channels. These channels include the structurally similar ether-à-go-go (EAG), EAG-related gene (ERG), and EAG-like channels (ELK), that are found in nematodes (*Caenorhabditis elegans*), fruit flies, rodents, humans, and other mammals (Pilot et al., 2003). The structural evolution of the K^+^ voltage-dependent channels has been addressed recently (Jegla et al., 2018). In particular, Jegla et al. (2018) explain how the structure of plant Kv channels makes them unique compared to the Kv channels of other organisms. The authors conclude that they have to be classified on their own. I come to the same conclusion, but from an entirely different position of the operational perspective. This point notwithstanding, a knowledge on plant Kv channels benefits from comparing structures and the associated functional properties.

The overall homologies among Kv channels aside, plant Kv channels lack the characteristic long cytosolic N-terminus of the Shaker channels that are known to bind to ancillary β-subunits (Hoshi et al., 1990; Schachtman et al., 1992; Long et al., 2005; Clark et al., 2020). These β-subunits are soluble proteins, essential for the function of the Shaker channels, and responsible for their so-called N-type inactivation (Figure 4D). N-type inactivation (autoinhibition) arises when the first 20–30 (largely positively charged) amino acids, which form a ball-like structure, physically enter the cytosolic end of the channel to plug and block ion entry to the channel pore. This process has been described as a “ball-and-chain” mechanism (Armstrong and Bezanilla, 1977; Zhou et al., 2001) and causes the closing of the channels some tens of milliseconds following channel activation with a change in membrane voltage. Plant Kv channels do not include such a domain and are not inactivated over time. The N-terminus is much shorter than typical of Shaker channels, but in some cases can interact with vesicle trafficking proteins, which introduces an additional level of regulation (see “SNARE proteins gate inward-rectifying Kv channels” section).

Plant Kv channels have an extended C-terminus, far longer than found among the Shaker subfamily (Figure 1A). This extended cytosolic domain generally comprises roughly half of the total sequence of residues and is recognized to be important for protein stability, tetramer assembly, and regulatory protein binding. The C-terminus contains, in order from the closest to pore domain to the furthest, the C-linker (see “Domain swapping and its implications for channel gating” section), the CNBD which is the typical domain carried by CNBD subfamily members, ankyrin domains, and an acid and hydrophobic residue domain (KHA; Very and Sentenac, 2003).

The CNBD domain is the typical domain found in cyclic nucleotide-gated (CNG) channels, hyperpolarization-activated cyclic nucleotide-gated channels (HCN), and ether-à-go-go type (KCNH) channels. CNG channels need this CNBD domain to bind to cyclic nucleotides (cGMP or cAMP depending on the channel) to be functional. The gating properties, maximal conductance, and kinetics of HCN channels are affected by the binding of cyclic nucleotides. Although KCNH channels do not depend on cyclic nucleotide binding, part of the protein mimics a binding pocket and incorporates a cyclic nucleotide. Plant Kv channels lack such a site mimic, but the CNBD is found in an “active” position by default, which precludes the need for cyclic nucleotide binding (Clark et al., 2020). In HCN channels, the CNBD domain rotates as part of channel gating leading to the open state (James and Zagotta, 2018). By contrast, the CNBD in plant Kv channels is critical for tetramerization (Daram et al., 1997; Dreyer et al., 2004). Some amino acids in the C-linker and CNBD of KAT1 (via the ^394^D-X-D motif) may be important for channel targeting to the plasma membrane, although tetramer stability has yet to be tested and could be compromised when changing these residues (Mikosch et al., 2006). Another region upstream of the KHA region seems to be important also for GORK tetramerization (Dreyer et al., 2004). The KHA region may contribute to channel clustering of the KST1 channel, the KAT1 homolog of potato, as clustering is lost when the KHA domain is removed (Ehrhardt et al., 1997).

Ankyrin domains are absent in KAT1 and KAT2 Kv channels. They consist of concave structure that is partially charged positive and another part is negative, at least in AKT1 channel (Sánchez-Barrena et al., 2020). These domains serve as binding sites for protein kinases, notably the CIPKs (Lee et al., 2007), and are described in “Calcium sensitive kinases and channels activation or inhibition” section.

### Diversity in plant Kv channels

**Figure 2:**
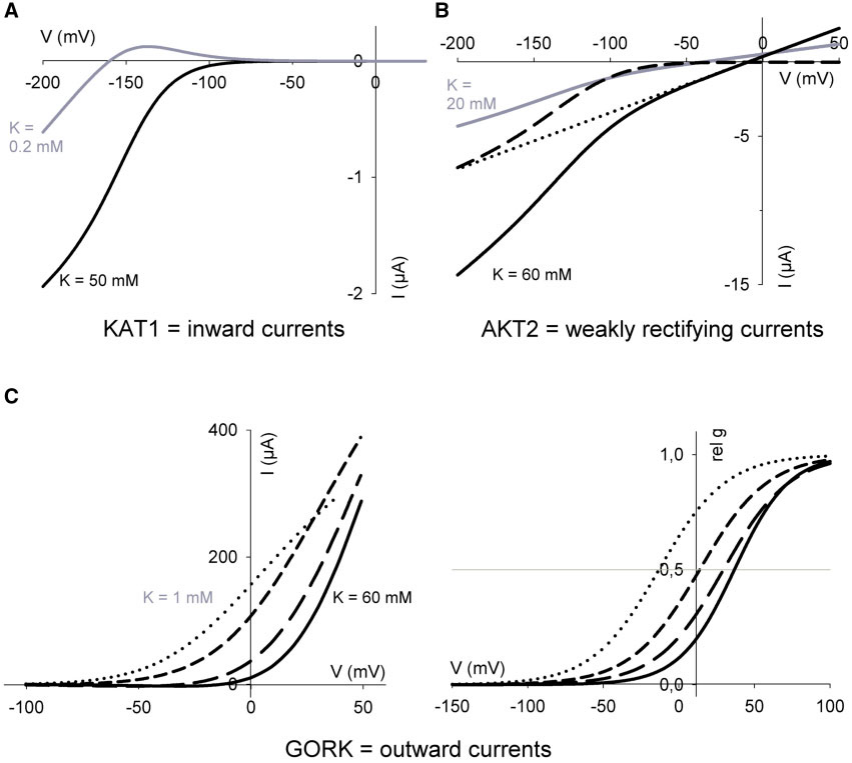
Representative steady-state current–voltage and conductance–voltage curves for the prevalent Kv channels in plants. Current–voltage (IV) curves are commonly described using the Boltzmann equation


Although plant Kv channels share a common structure, their properties differ substantially from one to another. Broadly speaking, the channels have been subdivided among five groups (Pilot et al., 2003). This classification is appropriate for flowering plants (angiosperms), but the distinctions between groups, for example, Groups I and II, are not obvious in some species, including fern, bryophyte, and gymnosperm clades (Dreyer et al., 2020). Apart from phylogeny, Groups I and II channels properties are too similar to be separated: they are activated by membrane hyperpolarization and facilitate K^+^ influx into the cell (Figure 2A; inward-rectifying channels AKT1, KAT1 and KAT2 in *Arabidopsis*; Schachtman et al., 1992; Gaymard et al., 1996; Pilot et al., 2001; Xu et al., 2006). Members of Group III are weakly regulated by voltage and are represented by the AKT2 channel in *Arabidopsis*. AKT2 remains partially open at all voltages and therefore mediates a “leak” of K^+^ subject to the electrochemical driving force on the ion (Figure 2B; Lacombe et al., 2000). The channels of Group V are activated by membrane depolarization and mediate K^+^ efflux (Figure 2C; outward-rectifying channels GORK and SKOR; Gaymard et al., 1998; Ache et al., 2000). Finally, Group IV channels are also referred to as “silent” channel subunits. This group is represented by KC1 in *Arabidopsis*. These channels are silent, because they do not form functional channel assemblies on their own, but function only in heterotetramers assembled with the other Kv channel subunits; in this context, they moderate the voltage sensitivity of channel gating (Duby et al., 2008).

(1) $${\text{I = g}}_{\text{max}}\left({\text{V-E}}_{\text{K}}\right)\text{/(1+exp}\left(\text{δ}\left({\text{V}}_{\text{1/2}}\text{-V}\right)\text{F/RT}\right)$$

**Figure:** which relates the current (I) to the electrochemical driving force as the difference in voltage (V) from the ionic equilibrium voltage (here for K^+^, E_K_) and from the voltage midpoint for channel activity (V_1/2_), the maximum ensemble conductance (g_max_), a voltage sensitivity coefficient or apparent gating charge (δ) that determines the steepness of the curve, and where F = Faraday constant, R = universal gas constant and T = temperature). The value of V_1/2_ also defines the mean energy needed to open the channel as the free energy


(2) $${\text{ΔG=-FδV}}_{\text{1/2}}$$

**Figure:** A, Current–voltage curves for the KAT1 channel. By convention, a negative current is defined as movement of positive charge into the cell. Currents in 50 mM K^+^ outside (*black line*) are scaled by 0.1 for comparison. As shown, the KAT1 V_1/2_ is −140 mV. This V_1/2_ is not sensitive to [K^+^] and, thus, as E_K_ is shifted to more negative voltages in 0.2 mM K^+^ (*grey line*) a small positive current appears at voltages positive of E_K_. The two relative conductance–voltage curves plotted are identical because E_K_ shift is not visible on this type of graph. The small positive current at low external [K^+^] is avoided in KAT1-KC1 heterotetramers, because the V_1/2_ values of these channels are shifted some 30–40 mV to more negative voltages. B, The weakly rectifying currents of AKT2 facilitate both K^+^ efflux (positive current) and influx (negative current), depending on the membrane voltage and E_K_. Current-voltage curves correspond to the situation in 60 mM (*black solid line*) and 20 mM K^+^ (*grey line*). The leak current of AKT2 (*dotted line*) is apparent as a current that activates instantaneously on voltage–clamp steps, separate from the steady-state currents (*black solid line*) and are described by the linear relation.


(3) $${\text{I=g}}_{\text{max}}\left({\text{V-E}}_{\text{K}}\right)$$

**Figure:**
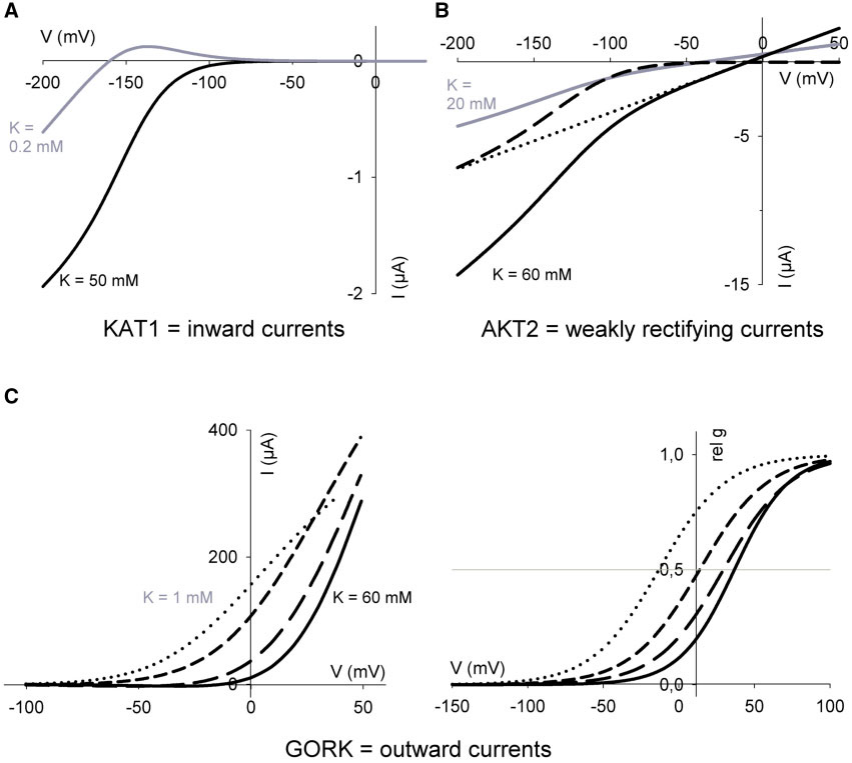
with the steady-state current described as a sum of components defined by Equations (1) and (3). The time-dependent component of AKT2 (*long dashed line*) can be extracted by subtracting the leak currents (*dotted line*) from the steady-state currents (*solid black line*). Note that, like KAT1, the value for V_1/2_ of AKT2 does not change with K^+^ concentration. C, Current– (*left*) and relative conductance–voltage (*right*) curves of GORK channel. The gating of GORK (also SKOR) is modified by the K^+^ concentration outside although K^+^ carrying the current comes from inside the cell. For the curves shown, the [K^+^] outside are 1 (dotted lines), 10 (short dashed lines), 30 (long dashed lines), and 60 mM (solid lines). The relative conductance–voltage curve avoids the visual bias of E_K_ displacement. Intersection with the horizontal grey line (*right*) marks the V_1/2_ values, showing that these shift to the right with [K^+^].

The different characteristics of Kv channels reflect the different physiological processes in which they are involved. AKT1 channels participate in K^+^ uptake in roots; the SKOR channel allows the efflux of K^+^ from cells around xylem to translocate the ion from root to shoot; K^+^ distribution in vascular tissues is mediated by AKT2, for example; and KAT1, KAT2, and GORK are vital for the effective kinetics of stomatal movement. KAT1 and KAT2 facilitate stomata opening by mediating K^+^ influx into the guard cell; GORK contributes to K^+^ efflux for stomata closing (Very and Sentenac, 2003; Gambale and Uozumi, 2006; Lebaudy et al., 2007).

## Voltage gating in plant Kv channels

### Up and down movement of the voltage-sensing domain

For Kv channels, membrane voltage is the most important stimulus and its action changes the conformational state of the channel. These conformational changes, generally referred to as gating, define the open or closed states of the channel.

**Figure 3:**
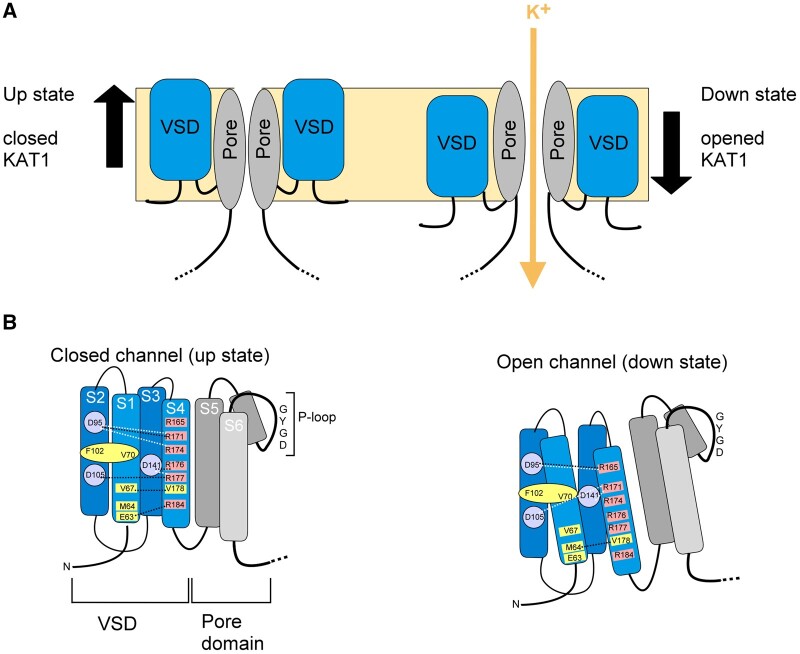
VSD movements of KAT1 between the open and closed states. A, Movement of the VSDs within the membrane between the “up” and “down” states and the corresponding channel activity for K^+^ permeation. B, Interaction between amino acid residues across transmembrane α-helices within the VSD, as implicated from crystallographic and residue substitution analyses of KAT1 with molecular dynamic simulations. Residues ^102^F and ^70^V (*yellow*, hydrophobic) form the hydrophobic core of VSD. Basic residues (*red*) that provide positive charge to the S4 segment and form salt bridges (*white lines*) with acid amino acids (*violet*). Salt bridges incorporate knowledge of KAT1 and general Kv channel structure (Lefoulon et al., 2014) and KAT1 crystal structure (*black lines*; Clark et al., 2020). Transition from the closed to open state realigns bridges and was identified through mutations F102W (F129W in KC1) that force the channel into the “up” state and D105E (D132E for KC1) that force the channel into the “down” state (Lefoulon et al., 2014).

All Kv channels harbor so-called voltage sensor domains (VSDs; Figure 1A). In general, VSDs are structurally conserved and consist of the N-terminal end and four transmembrane α-helices, designated S1–S4. The fourth VSD segment contains a repeated sequence of positively charged amino acids that are positioned across the electrical field of the membrane. As a consequence, the position in the membrane of this domain—and much of the rest of the VSD through its assembly—is driven by changes in the membrane voltage. When the electric field across the plasma membrane becomes more negative (hyperpolarization), the VSD moves downward toward the cytoplasmic face of the membrane, and similarly, as the membrane becomes less negative inside (depolarization) the VSD shifts away from the cytosolic side of the membrane. These two end-point conformations are generally referred to as the “down” and “up” positions, respectively. These movements are coupled through the linkage to the pore-lining helices to open and close the channel pore (Figure 3A). For example, the “up” position for the KAT1 channel represents the channel in its closed state while the “down” position represents the channel in its open state.

Significantly, how this movement impacts on gating differs between inward- and outward-rectifying channels. KAT1 is an inward channel that opens at voltages more negative than −100 mV. To test the VSD movement in KAT1, basic residues in the S4 helix of the VSDs were substituted with cysteines (Latorre et al., 2003). Cysteine residues react with methylthiocyanate compounds, including the water-soluble MTSET. Thus, the reactivity of the residues provides information about the accessibility of substituted amino acids to the aqueous solution on one side or the other of the membrane in the open and closed states. For example, specific amino acids are accessible to MTSET added outside, but they become inaccessible when the channel is stimulated by voltage to drive it into the open conformation. The simplest explanation, therefore, is that these amino acids move in and out of range of MTSET on the cytosolic side when KAT1 is closed and open, respectively (Latorre et al., 2003).

By contrast, SKOR is an outward-rectifying channel that opens with positive-going voltages (depolarization) from the K^+^ equilibrium voltage (E_K_; Figure 2C). SKOR is trapped in the open state by oxidizing agents, including H_2_O_2_ and MTSET, and this characteristic is observed when the channel is driven by voltage into “up” position (Garcia-Mata et al., 2010). Because hyperpolarization has opposite effects, closing outward-rectifying channels and opening inward-rectifying channels, we must conclude that VSD coupling to the pores differs between these two subsets of K^+^ channels.

It is of interest that some VSDs may act as channels in their own right. The Hv1/VSOP channel structure is very similar to the VSDs of Kv channels and incorporates a “proton wire” within the S1–S4 structure that conducts H^+^ across the membrane (Ramsey et al., 2006; Sasaki et al., 2006). Unlike Kv channels, Hv1 assembles functional channels as a dimer, the first subunit working as voltage sensor while the second subunit forms the pore. Functionality between voltage sensor and pore are interchangeable. The Hv1 VSD differs from the VSDs of the Kv channels, as it possesses an aqueous conduit in the center of the four transmembrane segments, allowing proton permeation (Ramsey et al., 2010). KAT1, by contrast, possesses a hydrophobic plug at the center of the VSD domain, formed by the union of the amino acids F102 and V70, which prevents H^+^ permeation (Lefoulon et al., 2014, Clark et al., 2020). Here the closed structure of the VSD is maintained by the formation of salt bridges between different positive and negative charged amino acids (Figure 3B).

### Domain swapping and its implications for channel gating

**Figure 4:**
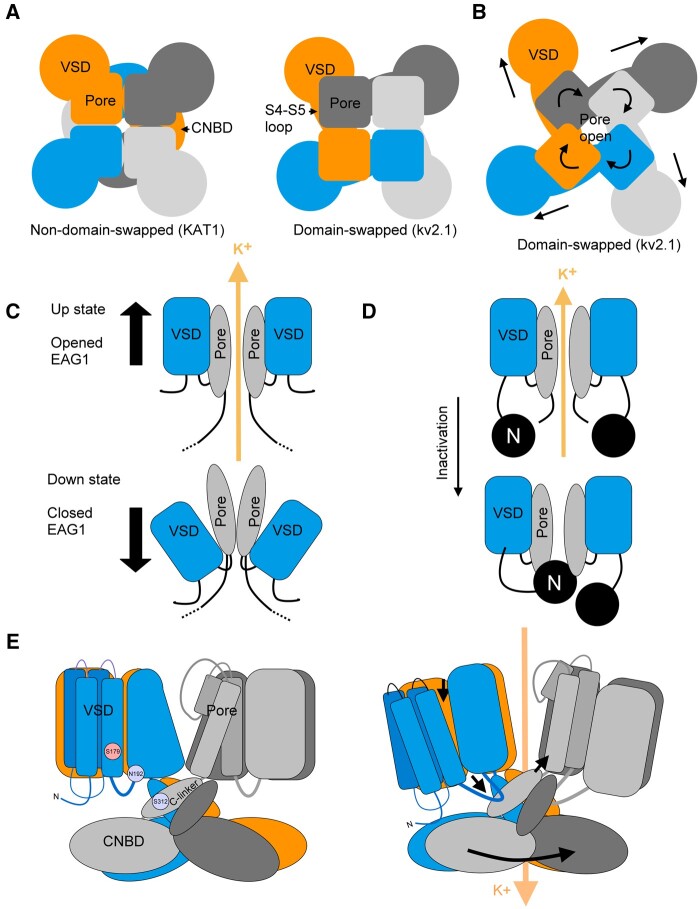
Gating mechanisms. A, Difference between nondomain-swapped and domain-swapped channels. Nondomain-swapped channels place the VSD adjacent to the pore domain of the same polypeptide subunit. Domain-swapped channels place the VSD adjacent to the pore domain of the neighboring subunit domain. The cytosolic S4–S5 linkage is much longer in domain-swapped channels. B, The concept of the iris diaphragm mechanism for domain-swapped channels, including the mammalian Kv2.1 channel. Here, VSD movement pulls on the S4–S5 linkage to rotate the pore domains and open the channel. C, The concept of the VSD plug is applied to nondomain-swapped channels such as the mammalian EAG1 channel. Here the channel opens with depolarization as the VSDs situate in the “up” state. When hyperpolarized, the VSDs situate in the “down” state and squeeze or “plug” access to the pore, thereby closing the channel. D, N-type inactivation engages a “ball-and-chain” mechanism in which charged groups located at the N-terminus are drawn into the pore when open and interact electrostatically to block further permeation. This mechanism gives rise to current inactivation in which the ensemble channel current rises and then decays as the channel pores are blocked. Deleting the first 20 residues of these channels abolishes this N-type inactivation. E, The mechanism of KAT1 channel gating deduced from crystal structure analysis depends on the S4–S5 loop of one subunit (*blue line*) pressing the C-linker of the neighboring subunit (*light grey*), displacing the C-linker to open the channel. The residues ^179^S, ^192^N, and ^312^S of KAT1 (^210^S, ^197^K, and ^329^S in AKT2) are critical for channel gating. From knowledge of HCN channels, it is likely that the CNBD domain rotates during channel opening.

How is the movement of Kv channel VSDs coupled to the opening and closing of the pore? And what determines whether the “down” position is coupled to channel opening or channel closing? The S4–S5 linkage between the VSD and pore domain appears to be critical. The mechanics of the mammalian Kv1-9 channels are best known from the crystal structures first resolved from Kv1.2 and Kv2.1. These channels have been described as “domain-swapped,” because the VSD domain of each subunit situates adjacent to the pore domain of the neighboring subunit (Figure 4A, right panel). In these channels, the gating mechanism gains leverage through this anchorage, enabling the VSD to pull on its pore domain through the linkage and dilating the channel pore, much like rotating the ring on an iris diaphragm to open a lens for light entry (Figure 4B, Long et al., 2005).

The structures of several CNBD channels—notably the human ERG, rat EAG1, human HCN1, the *L. licerasiae* LliK, and *C. elegans* TAX-4 channels, all from the KCNH, HCN, and CNG channel families—were resolved in the past few years (Whicher and MacKinnon, 2016; James et al., 2017; Li et al., 2017; Lee and MacKinnon, 2017; Wang and MacKinnon, 2017). By contrast, these channels display a “non-domain-swapped” structure in which each pore domain abuts on the VSD domain of the same polypeptide (Figure 4A, left panel). We now know that KAT1 shows much the same characteristics (Clark et al., 2020). It is most likely that other plant Kv channels will follow suit. Certainly for each of these channels, the S4–S5 linkages are relatively short by contrast with the mammalian Kv1-9 channels, including the *Shaker* channels.

The short-length linker of nondomain-swapped channels adds a physical constraint that argues against their functioning like domain-swapped channels. The short linkers cannot extend across the same distance and thus preclude VSD leverage with the neighboring pore domain. Lörinczi et al. (2015) and Tomczak et al. (2017) tested the relationship between VSD and pore domains by cutting the S4–S5 linkers of the rat EAG1 channel and coexpressing the split polypeptides in Xenopus oocytes. Their studies show that channel function is recovered when the linker is no longer intact. Even removing the S4–S5 linker entirely yielded channel currents that were similar to the currents of the intact EAG1 channel. These findings argue strongly against a mechanism relying on a direct physical coupling and “pull” through the VSD-pore domain linkage. Instead, the authors argue that the VSD may act as a plug that extends in down position below the inside of the pore to block K^+^ entry and efflux through the channel (Figure 4C).

The concept of a plug extension obviously cannot work with KAT1, because the “down” state of the VSD gives rise to an open KAT1 channel. Instead, the recent crystallographic analysis of KAT1 (Clark et al., 2020) suggests a toggle-switch mechanism that incorporates part of the C-terminal cytosolic domain. This domain incorporates a so-called C-linker comprising two α-helices in a hairpin shape located in the cytoplasm just below the S6 transmembrane α-helix (Figure 4E). The analysis suggests that when KAT1 VSD goes “down” with the membrane hyperpolarization, the intracellular loop between the S4 and S5 α-helices presses against the C-linker of the neighboring subunit, thereby moving the S6 α-helix to open the pore (Clark et al., 2020). Much the same mechanism has been proposed for gating of the HCN1 channel, which also opens when the VSD is in the “down” conformation and generates an inward current similar to KAT1 (Lee and MacKinnon, 2017). These findings give us a different perspective on the mechanics of KAT1 gating that may apply equally to other inward-rectifying K^+^ channels of plant. This point apart, they also show how little we know still about the mechanisms underlying gating of several Kv plant channels. Not least, although the GORK and SKOR channels are structurally similar to KAT1, the mechanism of their gating clearly cannot be understood as an opening by pressure on the C-linker with the VSD transiting to the “up” position.

### Differences between inward and outward gating

What defines the rectification of gating in the plant Kv channel subfamily? A strategy used by Gajdanowicz et al. (2009) and Riedelsberger et al. (2010) was to investigate the differences in sequence for the two channels by swapping domains one at a time to assess their importance for the gating control. Surprisingly, exchanging KAT1 and SKOR residues did not result in the exchange of channels gating properties but showed that gating could be modified, sometimes in unpredictable ways. Gajdanowicz et al. (2009) focused on differences between the S6 pore-lining α-helix. Using the voltage yielding half-maximal conductance, V_1/2_ (explained in Figure 2 legend) as a measure of gating showed that this value is displaced to more negative voltages when key residues in the S6 α-helix are swapped with those of GORK. These mutations induced a bias suppressing KAT1 opening. By contrast, introducing KAT1 residues to the SKOR channel pore domain introduced a leak current with a partial loss of voltage control of the channel (Riedelsberger et al., 2010). This behavior could be explained if the mutated SKOR channel was unable to close completely. One potential interpretation from these studies is that the pore diameter of KAT1 is wider than that of SKOR, but molecular dynamic simulations suggest this is not the case and that the vestibule leading into the KAT1 pore is narrower than that of SKOR. Thus, for now, we must conclude simply that the two pores are structurally different in the two types of channels.

Chimeras constructed with the rEAG1 and HCN1 (Kasimova et al., 2019) do shed some light on the problem of what determines the rectification of a channel. Molecular dynamic simulations and experimental analysis of these chimeras show that during the channel opening, negatively charged amino acids from α-helice S4 of HCN1 slide down and a break in hydrogen bonding forms at a serine residue in the middle of the S4 α-helix. This amino acid is absent in the EAG channels. Replacing this residue with a hydrophobic residue in the chimeric HCN1 with the rEAG1 pore domain reversed the rectification of the channel. If we align this region of the chimera with the plant Kv channels, it is clear that the consensus sequence is slightly different. The residues RLLRL of HCN1 are replaced with RLWRL for all inward plant Kv channels and with RLxRV for GORK and SKOR. Furthermore, an arginine is present at the HCN1 serine residue position in every case. Unquestionably, this region of the plant Kv channels will be of interest for future engineering.

### Kv channel gating by K^+^

One of the intriguing features of plant Kv channels is their dependence on K^+^ both as a permeant ion and as a gating ligand. For the outward-rectifying channels GORK and SKOR, but not for the inward-rectifying K^+^ channels, external K^+^ regulates channel activity. These channels, and their counterparts in other plant species (cf. Blatt, 1988; Blatt and Gradmann, 1997; Jezek and Blatt, 2017) represent the only known examples of enzymes for which K^+^ binding alters activity with a known mechanism. Increasing the K^+^ concentration outside has the effect of shifting V_1/2_ to more positive voltages roughly in line with the shift in E_K_ (Figure 2C). In effect, increasing K^+^ outside the cell favors the closed channel conformation so that the membrane voltage must be driven further to positive voltages in order to activate the channel. In guard cells, the interplay of K^+^ with voltage-dependent gating is crucial to ensure the capacity for stomatal closure across a wide range of external K^+^ concentrations (Blatt, 1988; Jezek and Blatt, 2017).

The mechanism for K^+^-dependent gating relies on K^+^ entry and occupation of the channel pore. Single site mutations of the SKOR channel in the TxGYG selectivity motif and residues abutting on the inside of the S6 α-helix lead to a reduced K^+^ sensitivity or a complete loss of K^+^ regulation in gating (Figure 1B; Johansson et al., 2006). Johansson et al. (2006) also showed that this sensitivity alters with depletion of K^+^ from within the pore when extracellular K^+^ is reduced. One interpretation is that K^+^ occupation at the pore entrance helps to stabilize the closed pore and increases the activation energy for movement of the VSD to the “up” position. This interpretation accords with much evidence that shows an, often tight coupling between the pore and the VSD in Kv channels (Palovcak et al., 2014). In short, constrictions of the pore region and pressure through the S6 α-helix mediate K^+^ sensing in these channels.

### Kv channel gating by protons

By contrast with GORK and SKOR, the gating of KAT1 and several other inward-rectifying K^+^ channels is affected substantially by H^+^ with increasing H^+^ concentrations outside facilitating KAT1 opening (Blatt, 1992). In the KAT1 homolog of potato, KST1, the ^267^H residue was suggested to be responsible for pH sensing (Hoth and Hedrich, 1999). However, subsequent work showed that an electron cloud of ^240^E, ^266^F, and ^267^H is responsible for the pH sensitivity of KAT1 (González et al., 2012). There is also evidence for KAT1 that the first extracellular loop between the S1 and S2 α-helices of the VSD and the cytosolic loop between the S2 and S3 α-helices are involved in external and internal H^+^ sensing, respectively (Tang et al., 2000; Wang et al., 2016). Thus, for example, exchange of the S1–S2 loop with that of the maize ZmK2.1 homolog, which is insensitive to external pH eliminates this pH sensitivity. In these instances, H^+^ affects not only the V_1/2_ for gating, but also activation and deactivation kinetics.

### AKT2 K^+^ leakage and gating

The AKT2 channel incorporates elements of gating of the inward-rectifying K^+^ channels and those of a leak-like conductance. As expected, the leak conductance responds near-instantaneously to changes in voltage, whereas the rectifying component of the AKT2 current exhibits slow activation kinetics and reaches a steady-state only after many tens of milliseconds. These two current components are separable based on their temporal characteristics so that subtracting the instantaneous component yields current–voltage curves with a modest voltage dependence (Figure 2B). The dual nature of AKT2 means that it can mediate both influx and efflux of K^+^, depending on the membrane voltage and K^+^ concentrations on the two sides of the membrane.

Three amino acids, distributed between the S4 and S5 loop and the C-linker, appear to determine the fractional balance in AKT2 current between the leak and voltage-dependent components; the latter is favored when these sites are mutated to alanine or asparagine (Michard et al., 2005; Figure 4E). Based on homology mapping to the structure of KAT1, these residues are closely juxtaposed on the cytosolic face of the membrane (Gajdanowicz et al., 2011; Clark et al., 2020). In KAT1, these residues are associated with the channel opening mechanics, and if mutated to alanine the channel becomes biased to the closed state (Clark et al., 2020). Here again, comparing these findings with our knowledge of EAG1 noted above, it is clear that the S4–S5 loop is important for voltage gating control.

The leak conductance of AKT2 plays an important role in the physiology of the plant. Mutating AKT2 channels to eliminate the voltage-dependent component yields a channel that is nonetheless sufficient to complement the *akt2* mutant phenotypes of slowed development and excitability in leaves vascular tissue. By contrast, the *akt2* phenotypes are not rescued by channels mutated to give only the voltage-dependent current component (Gajdanowicz et al., 2011, Cuin et al., 2018). Such differences are not surprising, as the voltage-dependent component, by nature, is specialized for activity over a limited range of physiological voltages.

Of interest, however, AKT2 is normally expressed in the phloem and the delayed development evident in the *akt2* mutant could be a consequence of altered transport, over longer distances, of metabolites such as sucrose (Deeken et al., 2002). Computational modeling suggests that switching between AKT2 gating “modes” of the leak and voltage-dependent conductances is sufficient to facilitate sucrose transport. How this facilitation is effected is more difficult to say. In general, all transport across a membrane is entangled through a common dependence on voltage as a driving force and as a consequence of ion transport. Kv channels are no exception and will influence other transport activities by altering the electrical balance across the membrane. It is likely that AKT2 is important for charge balance in conjunction with H^+^-coupled transport of sucrose. Regardless of the explanation, these two AKT2 mutations, and the knowledge of these domains in Kv channel gating, are clearly potential tools for manipulation and control plant growth.

### Regulation of voltage gating by heterotetramerization

One aspect of Kv channels is their assembly as tetramers to form functional channels and, as a consequence, the potential for mixed gating characteristics through the assembly of heterotetramers (Pilot et al., 2001; Xicluna et al., 2007; Lebaudy et al., 2010). Heterotetramers are widely found among the inward-rectifying channels and yield voltage dependencies that can vary depending on the subunit identity. Obviously, such variations can occur only over the longer timescales of channel gene transcription, translation, channel assembly, and export from the endoplasmic reticulum. A good example of differences with tetramer assemblies is KAT1 and KAT2. Channels assembled of KAT2 alone activate at very negative voltages, typically with V_1/2_ values near and more negative than −180 mV, whereas gating of KAT1 homotetramers yields currents with V_1/2_ values ∼40–50 mV more positive than KAT2 V_1/2_ values, and the gating of the heterotetramers falls between these two limits (Lebaudy et al., 2010). Analogously, heterotetramers of AKT2 and KAT2 show a reduced K^+^ leak conductance compared to the AKT2 homotetramer assembly, as well as an inverted sensitivity to pH and insensitivity to Ca^2+^ (Xicluna et al., 2007).

One other key example is that of KC1. Although this channel subunit fails to assemble functional channels on its own, KC1 heteromers with AKT1 are activated at substantially more negative voltages than is observed when AKT1 is expressed on its own. KC1 has a similar “inhibiting” effect on the other Kv channel subunits (Duby et al., 2008, Jeanguenin et al., 2011). The KC1 subunit is essential for K^+^ channel function nonetheless: *kc1* mutant plants wholly lack the inward-rectifying K^+^ current normally observed in *Arabidopsis* roots, suggesting roles in channel stability and, as noted below, in association with other regulatory proteins (Honsbein et al., 2009). Finally, there is some evidence that developmental regulation of KC1 expression is important for adjusting the gating characteristics of root tissues to conditions of low external K^+^ concentration (Figure 2A; Geiger et al., 2009).

### Regulation of voltage gating by clustering

One last mechanism of gating control, yet to be fully resolved, depends on Kv channel clustering. There has long been evidence that plant Kv channels congregate locally in clusters within the surface of the plasma membrane (Hurst et al., 2004; Meckel et al., 2004; Sutter et al., 2006; Sutter et al., 2007). For KAT1, such clustering is associated with channel traffic and is affected by signals such as abscisic acid (Hurst et al., 2004; Sutter et al., 2006; Sutter et al., 2007). Cell biological and electrophysiological analyses support the occurrence of these channels in assemblies of tens of channel tetramers that are studded across the cell surface.

In at least one instance, that of the GORK K^+^ channel, clustering is associated with gating. As with KAT1, the GORK channel is found in clusters of ∼600 nm diameter across the cell surface but, unlike KAT1, these clusters are stable at plasma membrane in the face of hormone exposure (Eisenach et al., 2014). However, the studies of Eisenach et al. (2014) show that GORK clustering is highly sensitive to the external K^+^ concentration, with clusters dispersing rapidly as the K^+^ concentration increases. Eisenach et al. also report that these changes in GORK clustering are blocked by external Ba^2+^ just as is gating, suggesting a link between gating and clustering. Indeed, we can imagine that interactions between the VSDs of GORK, when clustered, could be important for regulating channel activity by fostering or enhancing channel activity even at modest membrane voltages when external K^+^ is sufficiently reduced that the electrochemical driving force prevents its entry into the cell. Whether such behavior extends to other channels remains to be seen. The KST1 K^+^ channel was described to form clusters through its KHA domain. However, the KST1 current remains unchanged when KHA region is deleted (Ehrhardt et al., 1997).

## Kv channel gating regulation by ancillary proteins

### SNARE proteins gate inward-rectifying Kv channels

The plasma membrane is an interface of exchange, not only of ions and solutes via trans-membrane transport, but also of vesicular cargoes that deliver lipids, proteins, and other material to the membrane and secrete cargoes at cell surface into the apoplast. This traffic of membrane vesicles is driven by a complex machinery of proteins that maintain exchanges between subcellular compartments. Among these proteins, the Soluble NSF-Attachment protein REceptor proteins (SNAREs) drive the final steps in fusion of vesicles with their target membranes (Chen and Scheller, 2001). SNAREs assemble functional complexes of four discrete coiled-coil proteins defined by the core coiled-coil residues: the Qa-, Qb-, and Qc-SNAREs (or Qbc-SNARE) that normally localize to the target membrane, and a vesicular R-SNARE (Bassham and Blatt, 2008).

Conventional wisdom holds that membrane vesicle traffic affects ion transport over periods of many minutes to hours by modifying the population of channels and pumps at the cell surface. For example, abscisic acid induces KAT1 endocytosis with halftime of roughly 10 min (Sutter et al., 2007), and blocking exocytosis of the channel back to the plasma membrane slows stomatal opening over many tens of minutes (Eisenach et al., 2012). However, work over the past two decades has shown that SNARE proteins also regulate ion channel activities directly through physical interactions that take place with channels that are already present at the membrane.

The KAT1 channel is a prime example. Early studies of the Qa-SNARE SYP121 (=SYR1/PEN1) showed that direct injection of the SYP121^ΔC^ protein fragment (=Sp2 fragment), including the coiled-coil domain but lacking the membrane anchor, was sufficient to block abscisic acid regulation of the major K^+^ and Cl^−^ currents in tobacco (*Nicotiana tabacum*) guard cells, although the currents were still evident under voltage clamp (Leyman et al., 1999). Subsequent work (Honsbein et al., 2009;  2015; Grefen et al., 2010) showed that the inward-rectifying K^+^ channels KAT1 and KC1 bound selectively with SYP121, the binding altering the voltage-dependent characteristics of channel gating. Such changes in gating could not be explained as a consequence of vesicle traffic. Indeed, Grefen et al. (2015) showed that the interaction had a mutual effect with the SNARE altering gating and the channel enhancing SYP121-dependent vesicle traffic (see also Karnik et al., 2017).

**Figure 5:**
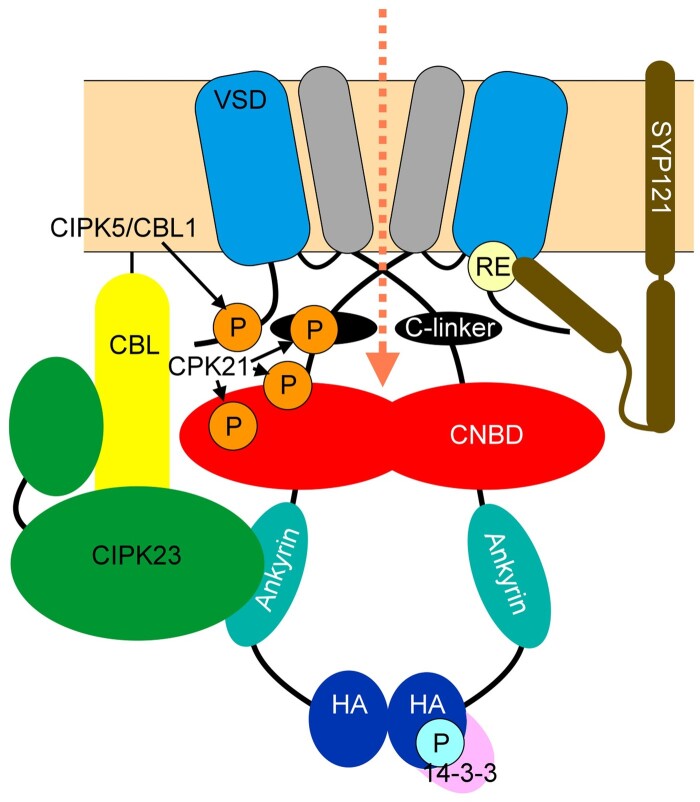
Kv channel regulation by interacting proteins and phosphorylation. Core KAT1 channel elements of the VSD (*blue*) and pore (*grey*) with the C-linker (*black*), CNBD (*red*), ankyrin domain (*turquoise*), and HA domain (*dark blue*). Interacting proteins include CBL and CIPK (here shown as CBL1 and CIPK23 in yellow and green) that associate with the ankyrin domain. 14-3-3 proteins (*pink*) interact with HA domain. Phosphorylation sites are indicated by circled letter P (in KAT1 ^676^S; in GORK ^344^T, ^518^S and ^649^S). The RYxxWE motif for SNARE binding is circled (RE) and is shown interacting with SYP121 to promote KAT1 activity.

We now know that, along with SYP121, the Qbc-SNARE SNAP33 and the R-SNARE VAMP721 bind to the channel VSD domain through a conserved RYxxWE motif located at the N-terminal cytosolic end of the channel and close to the S1 α-helix (Figure 5; Grefen et al., 2015; Zhang et al., 2015; Waghmare et al., 2019). Additionally, the channel binds with the regulatory protein SEC11, a member of the SEC1/MUNC18 protein family that regulates SNARE assembly and secretory traffic (Karnik et al., 2013 ,  2015; Waghmare et al., 2019). Of interest, the interaction of each of these SNAREs and SEC11 have different effects on channel gating, while the combination of the SNAREs in complex leads to a channel current that is little altered relative to the channels when expressed on their own (Waghmare et al., 2019). These findings, along with supporting isothermal calorimetry and Co-IP assays, lead to the conclusion that channel binding occurs in sequence with the several SNAREs during the vesicle fusion process.

**Figure 6:**
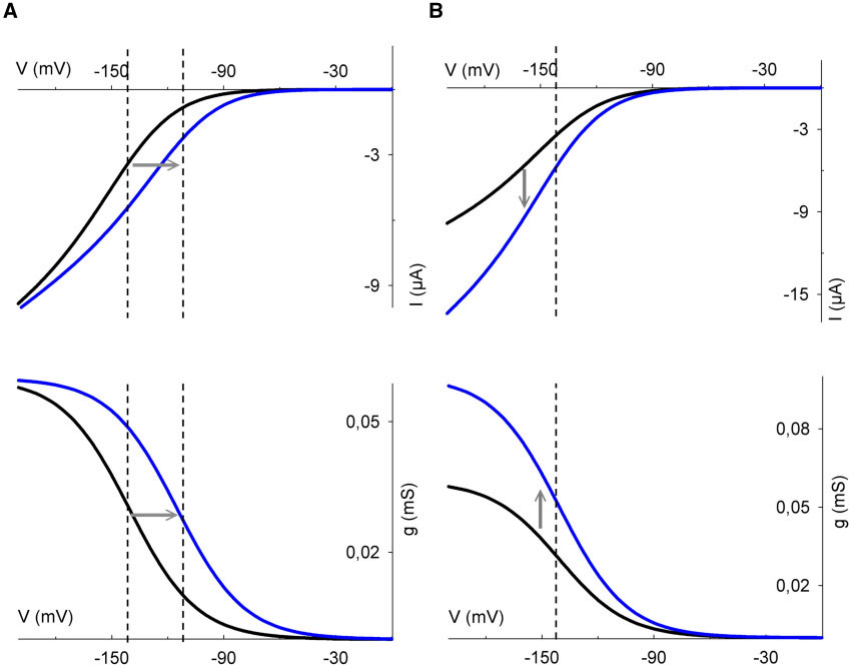
Effects of altered V_1/2_ and channel number on current–and conductance–voltage curves. Top panels are the current–voltage curves and bottom panels are conductance–voltage curves. In blue solid line is KAT1 gating changes provoked by an interacting protein. A, Change of V_1/2_ moves the curve to the right to increase the channel activity, for example, with KAT1-SYP121 interaction (KAT1 only *dark line*; KAT1 and SYP121 *blue line*). Note that VAMP721 would move the curve to the left. B, Increase of g_max_ moves the curve downward, reflecting a larger number of channels active at the membrane (*blue line*). Kinases and phosphatases typically affect the channel g_max_.

How might we understand SNARE binding and its influence on channel gating? Experimental evidence shows that SYP121 binding with KC1 (in complex with AKT1) and with KAT1 favors channel activation, displacing V_1/2_ to more positive voltages in each case (Honsbein et al., 2009; Grefen et al., 2010, 2015). For KAT1, SYP121 binding can be shown to favor the channel open lifetime (Lefoulon et al., 2018), effectively reducing the energy barrier for transition from the closed to the open state (Figure 6A). Of interest in this context, the KAT1 crystal structure (Clark et al., 2020) shows that RYxxWE motif interacts with the highly positive charged S4, especially through ^63^E (last residue of the motif) that forms hydrogen bonds with ^184^R of the S4 α-helix in the closed (“up”) position. A simple explanation, then, is that SNARE binding interferes with hydrogen bonding through ^63^E that otherwise would stabilize the channel in the close state, thus promoting channel opening (Figure 3B).

The interactions between these Kv channels and the SNAREs make physiological sense in the context of cell expansion. SNARE-channel interactions confer a mutual control on both solute uptake and exocytosis so that the increase in cell surface area and volume is coordinated with the increase in osmotic content. The importance of this coordination is evident from work with dominant-negative protein fragments that retain binding but are disrupted in their normal functions. Thus, the early studies with the SYP121^ΔC^ protein fragment showed that its expression allowed uncontrolled accumulation of osmotic solute, mainly K^+^ salts, and a highly significant increase in turgor (Sokolovski et al., 2008; Grefen et al., 2015). The complementary effect was observed on expressing the KC1 VSD alone (Grefen et al., 2015); in this case, the VSD promoted secretory traffic without enhanced K^+^ uptake, leading to a decrease in K^+^ and total osmotic content.

Analysis of several mutants yielded analogous findings. In roots, SYP121 and VAMP721 bind with KC1-AKT1 heterotetramers (Honsbein et al., 2009; Zhang et al., 2015). AKT1 dependent root growth was completely inhibited in the *syp121* mutant along with the K^+^ current, thus phenocopying *akt1* and *kc1* null mutant plants. By contrast, plants overexpressing VAMP721, which inhibits KC1-AKT1 activity, showed roots that grew only very slowly in low K^+^, much as is observed in the *kc1* mutant plants (Zhang et al., 2015). Finally, in stomata, SYP121^ΔC^ expression suppressed stomatal closure (Sokolovski et al., 2008) and the *syp121* mutation slowed stomatal reopening leading to plants that grew poorly, notably at low humidity and under high light. In each case, these characteristics are readily traced back to the coordination of the K^+^ currents with secretory vesicle traffic.

### 14-3-3 proteins modify Kv channel gating to prevent channel rundown

14-3-3 proteins are chaperones that bind specifically to phosphoproteins and regulate their function. They are involved in different signaling pathways, making the connection between environmental stimuli and protein regulation to provoke a physiological change (Denison et al., 2011). 14-3-3 proteins are known to regulate receptor kinases and other enzymes, to affect transcription factors including ABI5, and also to control the activities of H^+^-transporting ATPases and ion channels like TPK1, HvKCO1, and the Kv channels KAT1 and GORK (Baunsgaard et al., 1998; Sinnige et al., 2005; Van den Wijngaard et al., 2005; Latz et al., 2007). Several 14-3-3 proteins bind to the C-terminus of GORK in vitro when phosphorylated (Van Kleeff et al., 2018), and the outward-rectifying K^+^ current in root protoplasts is inhibited when exposed to barley 14-3-3 proteins (Van den Wijngaard et al., 2005). These effects appear limited to overall activity and do not affect the voltage dependencies of the channels. The effect of 14-3-3 proteins on inward-rectifying K^+^ channels contrasts with these observations: in this case, the 14-3-3 proteins appear to enhance the conductance and displace V_1/2_ to less negative voltages (Van den Wijngaard et al., 2005).

In general, the specificity of channels for 14-3-3 protein binding is weak. For example, KAT1 will also interact with endogenous 14-3-3 proteins of Xenopus when expressed in oocytes, and these interactions lead to the same enhancement and shifts in V_1/2_ as seen with the plant 14-3-3 proteins (Sottocornola et al., 2006, 2008). 14-3-3 proteins interact with the penultimate serine of KAT1 (Figure 5) to sustain KAT1 activity and prevent the loss of channel activity in isolation, what is often referred to as channel rundown (Hoshi , 1995). Channel activity can be sustained also if ATP is added to the bathing solution and through phosphomimetic mutation of the penultimate serine (S676D) of KAT1 or with the addition of fusicoccin which binds and stabilizes 14-3-3 interactions (Saponaro et al., 2017). From a physiological context, we can imagine that 14-3-3 proteins enhance and stabilize KAT1 activity in coordination with H^+^-ATPases thereby greatly enhancing K^+^ uptake. Indeed, it is known that H^+^-ATPases are activated by light, through phosphorylation and 14-3-3 protein binding (Kinoshita and Shimazaki, 2002; Inoue and Kinoshita, 2017). Proton fluxes promote the hyperpolarization that activates KAT1 and the channel activity is also supported by the binding of 14-3-3 proteins.

### Channel phosphorylation does not necessarily affect channel gating

While there is a large body of data on Kv channel regulation by elevated cytosolic-free Ca^2+^ concentrations ([Ca^2+^]_i_), we still have little idea in many cases about how this regulation is affected. For example, electrophysiological studies of guard cells have long shown that the inward-rectifying K^+^ channels are strongly inhibited by elevating [Ca^2+^]_i_ to 400–500 nM and above (Lemtiri-Chlieh and MacRobbie, 1994; Grabov and Blatt, 1997; Grabov and Blatt, 1999; Sokolovski et al., 2008). Thus, we don’t know if [Ca^2+^]_i_ acts directly on channels or is mediated secondarily through another Ca^2+^-binding protein. The actions of protein (de-)phosphorylation can affect the amplitude of [Ca^2+^]_i_ transients. As an example, we know that [Ca^2+^]_i_ spikes observed after challenging guard cells with nitric oxide are reduced by adding kinase antagonists to the medium (Sokolovski et al., 2005).

One certain fact is that [Ca^2+^]_i_ acts as a regulator of Kv channels through phosphorylation and may serve both upstream and downstream kinases. However, for Kv channels such actions are commonly associated with changes in the pool of activatable channels, in other words with an apparent change in the amplitude of channel conductance rather than with any changes in the voltage dependence or kinetics of channel activation (Figure 6B). The best-known example is in the activation of Ca^2+^-dependent protein kinases by [Ca^2+^]_i_. Two predominant families of kinases appear that respond to [Ca^2+^]_i_ and regulate Kv channels: the calcium-dependent protein kinases (CPKs) and CBL-interacting protein kinases (CIPKs). The latter kinases require the presence of calcineurin B-like (CBL)-binding proteins. CBLs chelate Ca^2+^ and then anchor and target CIPK to activate their target proteins for phosphorylation (Kudla et al., 1999; Shi et al., 1999; Halfter et al., 2000). One well-known example is the AKT1 channel, which requires the CIPK23/CBL1 or CIPK23/CBL9 pairs in vivo and when expressed in oocytes for activity (Table 1; Li et al., 2006). These kinases modify the channel maximum conductance, following a pattern of an “all or nothing” effect (Xu et al., 2006). However, AKT1 displays K^+^ currents when overexpressed in tobacco protoplasts, where the native CIPK/CBL kinase pair is likely to be active (Hosy et al., 2005).

**Table 1:** Binding and phosphorylation sites of kinases and phosphatases that interact with channels

Channel	Kinase	Binds to	Phosphorylation	Reference
AKT1	CIPK23/CBL*	Ankyrin domain via negative charges		Xu et al. (2006); Sánchez-Barrena et al. (2020)
	CIPK6/CBL*			Lee et al. (2007)
	CIPK16/CBL*			Lee et al. (2007)
	*CBL can be CBL1, CBL2, CBL3, or CBL9			
GORK	CPK21		Cytosolic C-terminus	Curran et al. (2011); Van Kleeff et al. (2018)
	CPK33			Corratgé-Faillie et al. (2017)
	CIPK5/CBL1		Cytosolic N-terminus	Förster et al. (2019)
	CPK13		Cytosolic N and C-terminus	Ronzier et al. (2014)
AKT2	CIPK6/CBL4		Modifies AKT2 traffic	Held et al. (2011)
**Channel**	**Phosphatase**	**Binds to**	**Phosphorylation**	**Reference**
AKT1	PP2CA	CIPK6		Lan et al. (2011)
	AIP1	CIPK6, 16 or 23		Lan et al. (2011)
	AHG1	CIPK6 or 16		(Lan et al., (2011)
	AIP1H	CIPK6, 16 or 23		(Lan et al., (2011)
AKT2	PP2CA	AKT2 Cytosolic C-terminus		Chérel et al. (2002)
GORK	PP2CA	GORK Cytosolic C-terminus		Lefoulon et al. (2016)
	ABI2	GORK Cytosolic N-terminus	GORK Cytosolic N-terminus	Lefoulon et al. (2016); Förster et al. (2019)

The activity of the GORK channel, similarly, is subject to regulation by phosphorylation and, again, the effect is separate from gating per se. GORK is functional in *Xenopus* oocytes and phosphorylation by CPK21 on residue ^649^S modulates GORK conductance (Curran et al., 2011; Van Kleeff et al., 2018; Figure 5, Table 1). The phosphomimetic S649E mutation increases GORK activity when expressed in *Xenopus* oocytes, but it does so without changing GORK gating properties (Lefoulon et al., 2016). Indeed, these studies showed that the density of channel protein with the wild-type GORK and GORK S649E mutant at the membrane was identical, despite the difference in activities. One simple conclusion, thus, is that phosphorylation acts as a switch to engage a population of GORK channels that would otherwise be inactive. However, to date, there remains a question about how elevating [Ca^2+^]_i_ acts to modify gating.

Phosphorylation can also affect channel traffic indirectly. The action of CIPK6/CBL4 is a good example, as the kinase pair interacts with AKT2 but does not phosphorylate the channel (Held et al., 2011). Instead, the CIPK6/CBL4 pair promotes AKT2 traffic, resulting in increase in channel population and conductance of AKT2 at the membrane. Here, again, the effect of kinases depends on Ca^2+^, but although its mode of action differs from that of CPK21 on GORK the consequence in ensemble channel activity is virtually the same.

## Conclusion

Mechanistic studies of the Kv channel family in plants have already added substantially to an understanding of their differing roles in plants, from nutrition to the regulation of stomata. These studies provide detail of the transport mechanics and its regulation and are now poised to help guide research toward engineering plants with improved efficiencies in nutritional control and water conservation. For example, the use of a chimera between the K^+^ channel Kcv, from *Chlorella* PBCV‐1 virus, linked to a photosensor module from the *Avena sativa* phototropin has enabled enhanced stomata opening and closing kinetics, thus improving plant growth under fluctuating light conditions (Papanatsiou et al., 2019). Kv channels are not only controlled by voltage, but also contribute to membrane voltage through the transport of K^+^. There are many examples of how Kv channels contribute to voltage, among these we know that the phenotypes of the double mutant of the H^+^-ATPases *aha1aha2* are largely rescued by elevating the K^+^ concentration in the growth media (Haruta and Sussman, 2012). Voltage deregulation of Kv channels has multiple physiological effect, for example, on phloem in affecting sugar transport and plant cell growth. Similarly, both experimental and model simulations of overexpression with channels and H-ATPases show that, modifying gating rather than Kv channel populations is the most effective way to manipulate cellular physiology (Wang et al., 2014a, 2014b). In short, understanding the biophysical and regulatory features of the Kv channels is opening opportunities for bioengineering (see “Outstanding questions” section). The next steps will be to develop networks of interacting transport proteins in order to access the complete sets of pathways essential to Kv channel regulation.

**Advances**

- Kv channel KAT1 was recently crystallised, revealing information about the channel opening mechanics.
- Gating of plant Kv channels has a distinct pattern from animal or bacterial gating.
- The VSD is highly conserved through all Kv channels independent of the organism.
- Movement of VSD controls vesicle traffic, and proteins involved in trafficking regulate Kv channel gating, not through controlling the total population of channels at the membrane.

**Outstanding questions**

- How can the GORK outward channel, a structurally similar channel to inward channel KAT1, work in reverse by being active when the membrane depolarises? What are the mechanics?
- Because Kv channels in plants are involved in plant cell growth and stomata aperture control, can gating be manipulated to enhance plant cell function to improve water use efficiency and growth?
- Does phosphorylation primarily regulate the population of available channels or can we find phosphatases or kinases that affect channel is voltage gating too?

## Notes

The author responsible for distribution of materials integral to the findings presented in this article in accordance with the policy described in the Instructions for Authors (https://academic.oup.com/plphys/pages/general-instructions) is: Cécile Lefoulon (cecile.lefoulon@glasgow.ac.uk).
